# Phenolic Fractions from *Vaccinium vitis-idaea* L. and Their Antioxidant and Anticancer Activities Assessment

**DOI:** 10.3390/antiox9121261

**Published:** 2020-12-11

**Authors:** Gabriele Vilkickyte, Lina Raudone, Vilma Petrikaite

**Affiliations:** 1 Laboratory of Biopharmaceutical Research, Institute of Pharmaceutical Technologies, Lithuanian University of Health Sciences, Sukileliu av. 13, LT-50162 Kaunas, Lithuania; lina.raudone@lsmuni.lt; 2 Department of Pharmacognosy, Lithuanian University of Health Sciences, Sukileliu av. 13, LT-50162 Kaunas, Lithuania; 3 Laboratory of Drug Targets Histopathology, Institute of Cardiology, Lithuanian University of Health Sciences, Sukileliu av. 13, LT-50162 Kaunas, Lithuania; vilma.petrikaite@lsmuni.lt

**Keywords:** lingonberry, phenolic compounds, antioxidant activity, anticancer activity

## Abstract

Lingonberry leaves and fruits are associated with a range of potential bioactivities related to their phenolic content and composition, but the identification of major biological activity markers remains limited. The present study aimed at the isolation of lingonberry phenolic fractions and biological activity evaluation of them. Crude dry extracts of lingonberry leaves and fruits were fractionated by chromatography using Sephadex LH-20 and analyzed by validated HPLC-PDA method. For each fraction, the anticancer activity against human clear cell renal cell carcinoma (CaKi-1), human colon adenocarcinoma (HT-29), and human malignant melanoma (IGR39) cell lines was determined using MTT assay, and the radical scavenging, reducing, and chelating activities were investigated using ABTS, FRAP, and FIC assays, respectively. Further, 28 phenolics were identified and quantified in the crude extract of lingonberry leaves and 37 in the extract of fruits. These compounds, during fractionation steps, were selectively eluted into active fractions, enriched with different groups of phenolics—monophenols, anthocyanins, phenolic acids, catechins, flavonols, or proanthocyanidins. Fractions of lingonberry leaves and fruits, obtained by the last fractionation step, proved to be the most active against tested cancer cell lines and possessed the greatest antioxidant activity. In this perspective, the predominant compounds of these fractions—polymeric and mainly A-type dimeric proanthocyanidins—also quercetin can be considered to be anticancer and antioxidant activity markers of lingonberries.

## 1. Introduction

*Vaccinium vitis-idaea* L. (lingonberry), spontaneous growing evergreen shrubs (family *Ericaceae* Juss.), are well known for their health-protective properties and potential use in the treatment of urinary tract infections and chronic diseases, associated with free radicals damage in the human body. Fruits and leaves of this plant are considered to be a good source of phenolic compounds, which ensure various mechanisms of the antioxidant defense system [1,2]. Major groups of lingonberry’s phenolic compounds are monophenols (prevalent in leaves), anthocyanins (prevalent in fruits), phenolic acids, flavonols, catechins, and proanthocyanidins, found in both raw materials [3,4]. Scientific studies highlight the chemotherapeutic and chemopreventive potential of phenolic compounds. One of the main advantages is that phenolic compounds can selectively inhibit cancer cell proliferation with no cytotoxicity on healthy cells. Low toxicity and multisignaling inhibitory effects are the key features in chemopreventive and chemotherapeutic strategies of phenolic compounds [5]. Phenolic compounds in combinations with chemotherapeutic drugs can act synergistically, sensitize resistant cancer cells, or prevent induced toxicities by decreasing reactive oxygen species (ROS) [6,7,8]. Phenolic compounds due to their structural peculiarities can not only directly scavenge free radicals but also modulate oxidative stress and redox status, which is crucially important in tumorigenesis and chemoresistance. Depending on conditions they are able to implement necessary chemotherapeutic antioxidant or pro-oxidant mechanisms [5,9,10,11].

A number of studies have been conducted on lingonberries’ raw materials phytochemical characterization and pharmacological evaluation, but there are challenging problems that arise in this domain [2,12,13,14]. One of the problems is that most studies examine the averaged biological activity of the lingonberry extracts, which is determined by the sum of synergistic and antagonistic interactions between all bioactive compounds. Also, extracts vary in phytochemical composition significantly. The contribution of individual phenolics or particular groups to the biological activity of lingonberries has not been extensively studied directly [15]. This complicates the identification of lingonberry’s biological activity markers. Moreover, crude plant phenolic extracts always contain sugars, pectins, proteins, and other impurities, which may limit the application and activity of the phenolics [16,17,18]. Another concern is the presence of thousands of compounds, complex and large phenolics structures, leading to difficult separation and quantification of them [19].

One way to overcome these problems is to purify or fractionate the crude extracts. The development of a fractionation methodology may improve the elucidation of the phytochemical and pharmacological characterization of lingonberry phenolics. Evaluation of the biological properties of fractions with prevailing groups of different phenolics is desirable for the selection of plants with increased levels of compounds that are pharmacologically relevant or for the development of functional food ingredients with health-protecting properties [20,21,22]. The advantage of using lingonberry’s phenolics as a source of antioxidants is that they are of greater benefit compared to synthetic ones due to their natural origin and possibly fewer adverse side effects on health [23,24].

An effective gel-filtration chromatography technique using Sephadex LH-20 was applied in the present material, resulting in three characteristic fractions from the crude extract of lingonberry leaves and three from fruits, and quantified using a novel developed and validated HPLC-PDA method. To the best of our knowledge, this is the first comprehensive report on the phytochemical composition of lingonberry leaves and fruits, revealing the presence of procyanidins B1, B2, B3, A1, A2, and C1. Antioxidant activity covering three distinct mechanisms of action, namely, free radical scavenging, reducing, and chelating activities in vitro were evaluated in order to provide a full picture of antioxidant activity capacity. Three human cell lines of different origins, namely, renal cell carcinoma (CaKi-1), human colon adenocarcinoma (HT-29), and human malignant melanoma (IGR39) were selected to test proliferation inhibitory activity of obtained lingonberry fractions and standard substances of predominant lingonberry phenolics. As far as we know, no study to date has compared the anticancer effect on these different cancer cell lines and free radical scavenging, ferric reducing, and chelating activities of lingonberry leaves and fruits after fractionation of phenolic compounds.

The aim of the present study was to determine the qualitative and quantitative profiles of obtained lingonberry fractions and to characterize their antioxidant activities and anticancer effect. Comprehensively characterized novel fractions with potential chemo-active phytochemicals could be further functionalized in the phytopreparations, functional food, or integrated into the phytochemotherapeutic strategies.

## 2. Materials and Methods

### 2.1. Chemicals and Solvents

HPLC grade standard substances—procyanidins B1, B2, C1, A1, A2, (+)-catechin, (−)-epicatechin, quercetin, quercitrin (quercetin-3-*O*-rhamnoside), isoquercitrin (quercetin-3-*O*-glucoside), avicularin (quercetin-3-*O*-arabinofuranoside), guaiaverin (quercetin-3-*O*-arabinopyranoside), rutin (quercetin-3-*O*-rutinose), reynoutrin (quercetin-3-*O*-xyloside), kaempferol, nicotiflorin (kaempferol-3-*O*-rutinoside), afzelin (kaempferol-3-*O*-rhamnoside), astragalin (kaempferol-3-*O*-glucoside), *trans*-cinnamic acid, caffeic acid, chlorogenic acid (3-*O*-caffeoylquinic acid), neochlorogenic acid (5-*O*-caffeoylquinic acid), cryptochlorogenic acid (4-*O*-caffeoylquinic acid), *p*-coumaric acid, ferulic acid, sinapic acid, benzoic acid, vanillic acid, protocatechuic acid, arbutin, and resveratrol—were purchased from Sigma-Aldrich (Steinheim, Germany), whereas cyanidin-3-*O*-galactoside, cyanidin-3-*O*-glucoside, cyanidin-3-*O*-arabinoside, procyanidin B3, hyperoside (quercetin-3-*O*-galactoside), and 6”-*O*-acetylisoquercitrin (quercetin-3-*O*-(6”-acetylglucoside)) were purchased from Extrasynthese (Genay, France).

Chemicals for antioxidant or anticancer activities assays—6-hydroxy-2,5,7,8-tetramethylchroman-2-carboxylic acid (Trolox), ethylenediaminetetraacetic acid (EDTA), 2,2′-azino-bis(3-ethylbenzothiazoline-6-sulfonic acid) diammonium salt (ABTS), 2,4,6-tri-(2-pyridyl)-S-triazine (TPTZ), ferric chloride (FeCl_3_), sodium acetate, 3-(2-pyridyl)-5,6-bis-(4-phenyl-sulfonic acid)-1,2,4-triazine (Ferrozine), 3-(4,5-dimethylthiazol-2-yl)-2,5-diphenyltetrazolium bromide (MTT)—were obtained from Sigma-Aldrich (Steinheim, Germany), whereas potassium persulfate (K_2_S_2_O_8_), anhydrous ferrous chloride (FeCl_2_) were from Alfa Aesar (Karlsruhe, Germany). All standard substances and chemicals were of analytical grade.

The plasticware for cell cultures was obtained from Techno Plastic Products (Trasadingen, Switzerland), Corning (Corning, NY, USA), and Thermo Fisher Scientific (Waltham, MA, USA). Dulbecco’s modified Eagle high glucose medium (DMEM Glutamax), TrypLE^TM^ Express reagent, fetal bovine serum (FBS), penicillin/streptomycin solution (100×), and phosphate-buffered saline (PBS) were purchased from Gibco (Thermo Fisher Scientific Inc., Waltham, MA, USA).

The following solvents were used: 99.9% acetonitrile, 99.9% acetone, 99.9% methanol, 99.9% dimethyl sulfoxide (DMSO), 99.8% anhydrous acetic acid, 37% hydrochloric acid (HCl), all of which were obtained from Sigma-Aldrich (Steinheim, Germany); 99.8% trifluoracetic acid—from Merck (Darmstadt, Germany); and 96% ethanol—from Vilniaus degtine SC (Vilnius, Lithuania). The water used in this research was purified on a Millipore Milli-Q apparatus.

### 2.2. Cell Cultures

Human malignant melanoma cell line (IGR39), human colon adenocarcinoma cell line (HT-29), and human clear cell renal cell carcinoma line (CaKi-1) were obtained from American Type Culture Collection (ATCC, Manassas, VA). Cancer cell lines were cultured in DMEM Glutamax medium supplemented with 10% FBS and 1% antibiotics at 37 °C with 5% CO_2_ in a humidified atmosphere. Cell cultures were grown to 70% confluence, trypsinized with 0.125% TrypLE™ Express solution before passage, and used until passage 20.

### 2.3. Plant Material

Plant raw materials for research were randomly collected at the end of the fruits ripening period in September 2019 in North-East Lithuania (in the temperate climate zone and the sub-region of Atlantic-European continental, predominant mixed and broad-leaved forests) from different natural sites of lingonberries (56°00′40.6″ N 25°31′29.4″ E, 55°59′20.6″ N 25°25′48.2″ E, 56°04′32.8″ N 25°29′41.1″ E, 55°35′45.1″ N 26°07′53.7″ E, 55°39′59.3″ N 25°59′26.7″ E (WGS)) and then pooled. Fruits of lingonberries were immediately lyophilized in a ZIRBUS sublimator 3 × 4 × 5/20 (Bad Grund, Germany) in 0.01 mbar, −85 °C conditions, whereas leaves were dried at room temperature in a dark place. Freeze-dried fruits and air-dried leaves were ground with a Retsch 200 mill (Haan, Germany) to homogenous powder.

### 2.4. Production of Dry Extracts

Experimentally selected extraction conditions, leading to the highest (*p* < 0.05) yields of phenolics, determined by the HPLC-PDA method, were as follows. A precise weight of 40 g of powdered lingonberry leaves was extracted with 250 mL of 80% aqueous acetone, and the same precise weight of powdered lingonberry fruits was extracted with 200 mL of 70% aqueous acetone by ultra-sonication in Elmasonic P ultrasonic bath (Singen, Germany) for 15 min. The extraction procedures were repeated four times for leaves, and three times for fruits, until complete exhaustion. Supernatants of each raw material were combined after centrifugation (3000× *g*, 10 min) in a Biofuge Stratos centrifuge (Hanau, Germany). Obtained extracts were evaporated in an IKA RV 10 rotary evaporator (Staufen, Germany) at 40 °C until the acetone was removed and subjected to lyophilization to get dry extracts of lingonberry fruits and leaves.

### 2.5. Isolation of Phenolic Fractions by Column Chromatography

**Figure 1:**
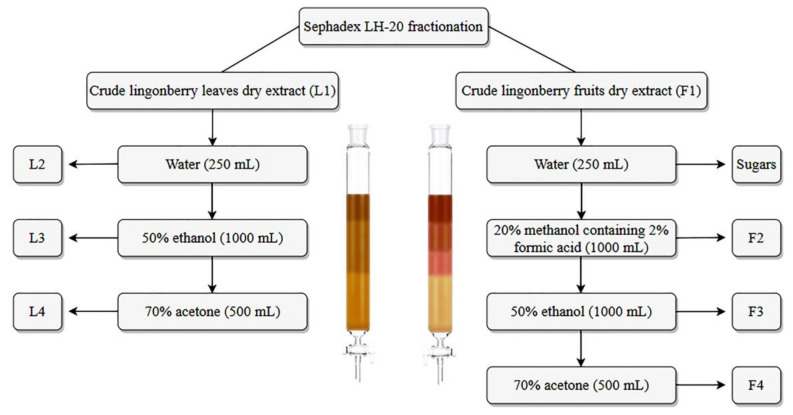
Scheme illustrating the fractionation sequence of extracts of lingonberry fruits and leaves.

For fractionation, 5 g portions of dry extracts of lingonberry leaves and fruits were dissolved in 200 mL of 50% methanol and applied to a glass column (3 × 60 cm) packed with already equilibrated gel filtration resin, Sephadex LH-20 (GE Healthcare Biosciences, Uppsala, Sweden), which was used as a stationary phase. Various elution ways were tested by using different amounts of water, different concentrations of ethanol, methanol, and acetone aqueous solutions as a mobile phase on the basis of previous researches [22,25,26]. The point at which the solvent was changed was according to the HPLC-PDA profile of collected solutions. The purest, highest yield phenolic fractions from lingonberry leaves (marked L with fraction number) and fruits (marked F with fraction number) were achieved when washing crude extracts according to the scheme in Figure 1. The fractionation procedures were repeated up to four times to collect sufficient amounts of fractions. Obtained fractions were rotary evaporated under reduced pressure and the resulting residue freeze-dried to get dry fractions, which were stored at −20 °C until analysis. All obtained further results were re-calculated for the dry weight (DW) of dry crude extracts or obtained fractions.

### 2.6. HPLC-PDA Analysis

Phenolics in crude extracts and each fraction of lingonberries were identified and quantified by HPLC-PDA (Waters e2695 Alliance system, Waters, Milford, MA, USA) according to Raudone et al. with some modifications [27]. Chromatographic separation was carried out on an ACE C18 reversed-phase column (250 mm × 4.6 mm, particle size 3 µm; ACT, UK) with a gradient elution consisting of 0.1% trifluoroacetic acid in water (eluent A) and acetonitrile (eluent B) at a flow rate of 0.5 mL/min, injection volume of 10 µL, and column temperature maintained at 35 °C. The gradient pattern was 0 min, 10% B; 0–40 min, 30% B; 40–60 min, 70% B; 60–64 min, 90% B; 64–70 min, 10% B. Prior to HPLC-PDA analysis, all samples were dissolved in 70% ethanol until complete dissolution, obtaining a concentration of 1 mg/mL, and filtered through a pore size 0.2 µm PVDF syringe filters (Macherey-Nagel GmbH & Co. KG, Düren, Germany).

The described modified method was validated following international guidelines [28]. The selectivity of peaks was evaluated and phenolic compounds were identified by comparing the retention times, UV spectra of the analytes with those of the reference compounds, and on the basis of previous reports on lingonberry phenolics. The PDA detector was set at a wavelength of 280 nm for proanthocyanidins and catechins, 360 nm for flavonols, 520 nm for anthocyanins, 330 for hydroxycinnamic acids, and 260 nm for hydroxybenzoic acids. All phenolics were quantified according to 5–7 points linear calibration curves of external standards, except well-known predominant compounds of lingonberry leaves—quercetin-3-*O*-(4”-(3-hydroxy-3-methylglutaryl)-rhamnoside (quercetin-HMG-rhamnoside), and 2-*O*-caffeoylarbutin, because of commercially unavailable standards. They were tentatively quantified using calibration curves of standard substances with similar chemical structures. Limits of detection (LOD) and of quantification (LOQ) were determined via the signal-to-noise ratio method. The trueness of the method was expressed as percent recoveries of phenolics at low, medium, and high concentrations of range, each analyzed in triplicate. To assess the repeatability and intermediate precision of the method, relative standard deviation percentages (% RSD) of peak areas of each quantified phenolic compound were calculated within (six times per day) and between days (three consecutive days), respectively, resulting in total repeatability of 18 replicates.

### 2.7. Antioxidant Activity Assays

Antioxidant activities of the crude extracts and fractions of lingonberries were evaluated by three different in vitro spectrophotometrical assays to determine the most active lingonberry phenolics and compare their predominant mechanisms on the antioxidant defense system. For antioxidant activity estimation, all testing samples were dissolved in 70% ethanol until complete dissolution, obtaining a concentration of 0.2 mg/mL.

ABTS radical scavenging activity was evaluated by the method described by Re et al. [29]. Briefly, 2 mM ABTS radical cation was formed by a reaction between ABTS aqueous solution and K_2_S_2_O_8_ in darkness at room temperature for 16 h. The obtained stock solution was diluted with distilled water for the absorbance of 0.80 ± 0.02 at 734 nm, determined by spectrophotometer (Spectronic CamSpec M550, Garforth, UK). The working ABTS solutions (3 mL) were mixed with test solutions—crude extracts or fractions of lingonberries (20 µL)—and the absorbance of these mixtures was measured at 734 nm after 1 h of incubation. The results were expressed as micromolar of standard antioxidant—Trolox equivalents per gram of dry weight of fractions (µM TE/g DW).

In the case of reducing activity, working FRAP solutions (3 mL), consisting of 300 mM acetate buffer, 10 mM TPTZ in 40 mM HCl, and 20 mM FeCl_3_ in a final ratio of 10:1:1 (*v*/*v*/*v*), were mixed with test solutions (20 µL), and the absorbance was recorded at 593 nm after 1 h of incubation at room temperature [30]. The ability to reduce ferric ions was expressed as Trolox equivalents per gram of dry weight of fractions (µM TE/g DW).

To assess chelating activity, testing solutions (1 mL) were mixed with 2 mM FeCl_2_ (50 µL), followed by adding 5 mM ferrozine solution (0.2 mL), incubation for 10 min at room temperature, and absorbance recording at 562 nm. Blank and negative control solutions were prepared in the same manner, only ferrozine and testing solutions, respectively, were substituted by the solvent [27]. Micromolar of strong chelating agent—EDTA equivalents per gram of dry weight of fractions (µM EDTA/g DW)—was the measurement unit.

### 2.8. Cell Viability Assay

The effect of lingonberry fractions and standard substances of phenolic compounds on cancer cell viability was studied using an MTT assay described by Grigalius and Petrikaite [31]. Briefly, 100 μL of cells were seeded in 96-well plates in triplicate (5 × 10^3^ melanoma and renal cancer cells and 8 × 10^3^ colorectal carcinoma cells per well) and incubated at 37 °C. After 24 h of incubation, serial double dilutions of lingonberry fractions, including crude extracts, and standard substances of phenolic compounds were made in microplates (standard substances were dissolved in DMSO). Cells treated only with the medium (or medium with 0.5% of DMSO in the case of standard substances) were used as a negative control, whereas medium without cells served as a positive control. After 72 h of incubating at 37 °C, the medium in all the wells was replaced with the fresh one containing 0.5 mg/mL of MTT. The liquid was aspirated from the wells and discarded after 3–4 h. Formazan crystals were dissolved in 100 μL of DMSO, and absorbance was measured at 570 nm (test wavelength) and at 630 nm (reference wavelength) by multi-detection microplate reader Multiskan GO (Thermo Fisher Scientific Oy, Ratastie, Finland). Hill fit to lingonberry fraction or standard substance dose–cell metabolic activity (absorbance) curves were applied and results of cell viability assay were expressed as the effective concentration (EC_50_) values, which reduce cell viability by 50%.

### 2.9. Statistical Analysis

All data were given as mean ± standard deviation (SD) from at least three replicates for each sample. In order to determine significant differences between values, analysis of variance (ANOVA) followed by a Tukey post-hoc test was performed. The significance of the difference was defined at the 5% level (*p* < 0.05). Principal components analysis (PCA) was implemented to highlight differences and classify obtained fractions of lingonberry. Correlations between variables were analyzed using Pearson’s correlation test. Data were processed using IBM SPSS Statistics version 26.0 package and Microsoft Office Excel 2016 software.

## 3. Results

### 3.1. HPLC-PDA Method Validation Parameters

Linear profiles (*r*^2^ > 0.999) of the response versus concentration of each phenolic compound were achieved at minimum ranges of 1.6–50.0 µg/mL (for the majority of compounds) and a maximum range of 31.3–500.0 (for arbutin). LOD and LOQ of all tested phenolics varied in the range of 0.03–0.90 μg/mL and 0.08–2.67 μg/mL, respectively. Obtained intra-day and inter-day precision values (expressed as % RSD) were lower than the 2% threshold, with total repeatability of different phenolics varying from 0.1 to 1.7% (Supplementary Table S1). The percentage recoveries of tested phenolics were 91.8–107.7%, 90.1–106.9%, and 90.3–106.2% at low, medium, and high concentration levels, respectively, with the mean value of trueness—100.1%, and RSD—1.0% (Supplementary Table S2).

### 3.2. Qualitative and Quantitative Analysis of Phenolics

**Figure 2:**
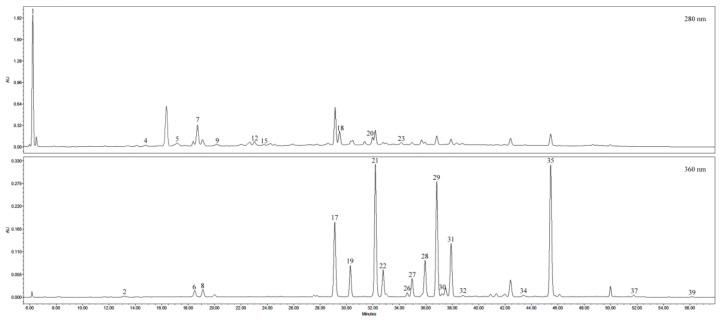
HPLC-PDA profiles at 280 nm and 360 nm, showing phenolics separation in the crude extract of lingonberry leaves. Peak assignments correspond to Figure 3 and are indicated in Table 1.

Qualitative analysis of a crude dry extract of lingonberry leaves led to the identification of 28 phenolic compounds, belonging to subgroups of monophenols, flavonols, catechins, proanthocyanidins, and phenolic acids (Figure 2). Obtained chromatograms, distinguished by the complexity of different groups of phenolics, confirmed the necessity of fractionation of crude extracts.

The results of the quantitative analysis provided in Table 1 demonstrate the contents of phenolic compounds in different fractions, including crude extract, of lingonberry leaves. Assessing each fraction composition, it was observed that crude extract of lingonberry leaves (L1) can be characterized by high levels of arbutin (made up 53.1% of the total identified phenolics), followed by (+)-catechin, procyanidins A1, B3, 2-*O*-caffeoylarbutin, and (–)-epicatechin. Large amounts and diversity of flavonols were also observed. Compounds of this group contributed up to 11.9% of the total identified phenolics in the crude extract of lingonberry leaves, with avicularin, hyperoside, and quercetin-HMG-rhamnoside being predominant. From the results, it is clear that flavonols of lingonberry leaves consisted mainly of the glycosides of quercetin (97.3%), whereas glycosides of kaempferol and aglycones of flavonols were not prevalent in L1. In the profile of flavonol glycosides, rhamnosides and arabinosides were predominated (32.1% and 28.1%, respectively). Phenolic acids—prevailing chlorogenic and cryptochlorogenic acids and lower (*p* < 0.05) amounts of neochlorogenic and *p*-coumaric acids—contributed only 2.7% of the identified phenolics content and can be considered as minor phenolics of lingonberry leaves.

The fraction of lingonberry leaves marked L2 was distinguished by a rather simple profile of phenolic compounds, basically consisting of arbutin (97.5% to total identified phenolics content) and much lower (*p* < 0.05) amounts of hydroxycinnamic acids. These compounds were effectively isolated with this fraction at the first fractionation step. As the main groups of phenolic compounds of fraction L3, flavonols, and catechins, as well as their derivatives—B-type dimeric proanthocyanidins (distributed in a ratio of 3.3:4.7:2)—can be regarded. Out of these groups, the highest (*p* < 0.05) amount of (+)-catechin was determined. The purest fraction of lingonberry leaves, marked L4, was achieved during the last fractionation step and distinguished by high contents of polymeric and mainly A-type dimeric proanthocyanidins. Procyanidin A1 contributed 52.8% to the total identified phenolics of this fraction and thus can be considered as the predominant phytochemical marker.

Overall, each of the fractions differed in their content of phenolic compounds and prevailing phytochemical markers. The total sum of identified phenolics arranged in the following ascending order: L4 < L1 < L2 < L3, and confirmed the hypothesis that fractionation may enhance levels of major phenolic compounds by concentrating them during washing steps.

**Figure 3:**
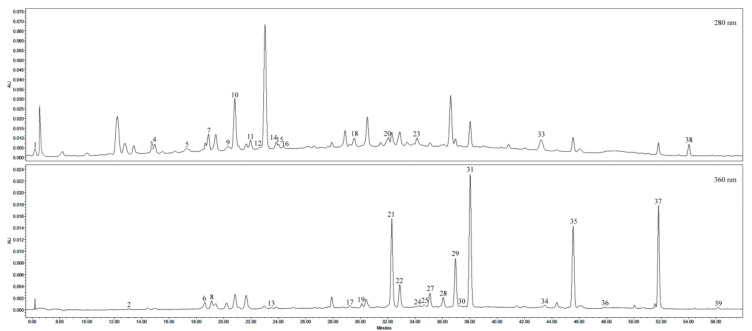
HPLC-PDA profiles at 280 nm and 360 nm, showing phenolics separation in the crude extract of lingonberry fruits. Peak assignments correspond to Figure 2 and are indicated in Table 2.

The phenolic profile of crude dry extract of lingonberry fruits basically comprised of anthocyanins, phenolic acids, monophenols, flavonols, catechins, and proanthocyanidins (Figure 3). Thirty-seven chromatographic peaks were identified as phenolic compounds.

The distribution of phenolic compounds in the crude dry extract and fractions of lingonberry fruits is shown in Table 2. Matrix of crude lingonberry fruits marked F1 was predominated by proanthocyanidins (24.6% to total phenolics content) and flavonols (20.6% to total phenolics content) with the highest (*p* < 0.05) amounts of procyanidins A1, B2, and quercitrin out of these groups. Flavonols in fraction F1 included quercetin derivatives (83.1%), relatively high amounts of quercetin aglycone (16.1%), and much lower (*p* < 0.05) contents of kaempferol derivatives. Profile of flavonols glycosides consisted of rhamnosides (52.3%), galactosides (14.7%), arabinosides (11.8%), and more than twice lower presence of other sugar moieties. Benzoic acid, followed by high levels of (+)-catechin, which made up 15.4% and 13.8%, respectively, of the total identified phenolics content, can be considered as phytochemical markers of F1. Other groups of phenolics—anthocyanins, hydroxycinnamic acids, and monophenols—were not prevalent in the obtained crude extract of lingonberry fruits.

The fraction of lingonberry fruits marked F2 did not contain flavonols, catechins, proanthocyanidins, and was comprised of anthocyanins (40.8%), phenolic acids, including their precursors (56.5%), and a small amount of arbutin. Cyanidin-3-*O*-galactoside, benzoic, and chlorogenic acids were regarded to be predominant phytochemical markers of this fraction. Major phenolics of fraction F3 were (+)-catechin, also quercitrin, procyanidin B1, and hyperoside. Generally, this fraction was characterized by high levels of flavonols, catechins, and B-type dimeric proanthocyanidins (contributed 48.8%, 31.1%, and 18.8%, respectively, of total identified phenolics). It may be noted that fraction F3 was enriched with a small amount of stilbenoid resveratrol (comprised less than 1% of total phenolics). The last fraction of lingonberry fruits, marked F4, was distinguished by the highest (*p* < 0.05) content of procyanidin A1, followed by procyanidin A2, and accounted for 34.5% and 25.1%, respectively, of total identified phenolics. Besides proanthocyanidins, high levels of quercetin and lower levels (*p* < 0.05) of kaempferol were also observed; the sum of them made up 8.7% of the identified phenolics content in this fraction.

Concerning the total amounts of detected phenolic compounds within different fractions of lingonberry fruits, the following ascending order was determined: F1 < F4 < F2 < F3. Contents of phenolics in the crude extract of lingonberry fruits—F1—were even 24 times lower, compared with the most abundant fraction of lingonberry fruits—F3.

### 3.3. Principal Component Analysis of Phenolics in Lingonberry Fractions

To classify fractions of lingonberry leaves and fruits, PCA was performed using relative amounts of the phenolic compounds as percentages of the total content of each fraction. The three principal components explaining 97.4% of the total data variance were obtained. The PC1 accounted for 56.2% of the total variance and positively correlated with procyanidins B1, B2, B3, (+)-catechin, (−)-epicatechin, quercitrin, hyperoside, avicularin, quercetin-HMG-rhamnoside, rutin, reynoutrin, and other flavonol glycosides. The second PC2 component described 32.5% of the total variance and differentiated samples containing the highest levels of arbutin, cyanidin-3-*O*-galactoside, cyanidin-3-*O*-glucoside, cyanidin-3-*O*-arabinoside, also chlorogenic, cryptochlorogenic, protocatechuic, *p*-coumaric, and other phenolic acids. The last PC3 component constituted the lowest part of the total variance (8.7%) and highly correlated with positive loadings of procyanidins C1, A1, A2, and quercetin.

**Figure 4:**
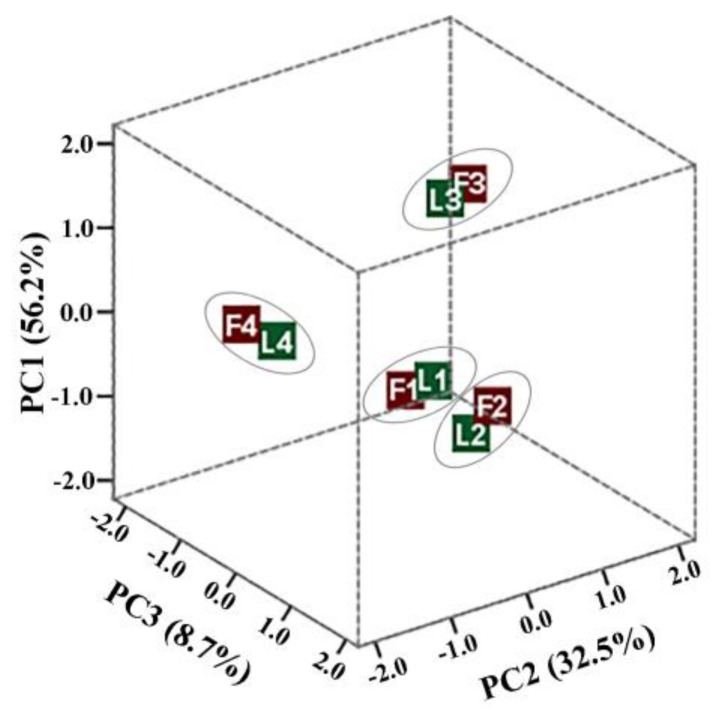
Score plots for principal components obtained by PCA based on relative quantification data of the fractions of lingonberry leaves and fruits.

The score plots of lingonberry fractions showed their arrangement into the four groups according to closeness with principal components (Figure 4). The first group was located at similar distances of all principal components and included crude dry extracts of lingonberry leaves and fruits, marked L1 and F1, respectively. This group was characterized by the highest diversity of phenolic compounds. The second group was located at the positive side of PC2 and coupled fractions marked L2 and F2, which were obtained from the first fractionation steps. This group was distinguished by high amounts of arbutin (in the case of leaves fraction), phenolic acids, and anthocyanins (in the case of fruits fraction). F3 and L3 fractions, which were obtained from lingonberry fruits and leaves, respectively, as a result of elution with 50% ethanol, were distanced from all other samples and were grouped at the positive side of the PC1, thus indicating their richness of catechins, B-type dimeric proanthocyanidins, and flavonols. The fourth group, which was located at the positive side of the PC3, included F4 and L4 fractions, obtained from the last fractionation step with 70% acetone, and was distinguished by the highest amounts of polymeric and mainly A-type dimeric proanthocyanidins, also flavonol aglycones.

The results of PCA revealed that the phenolic profile of investigated fractions significantly differed from each other in the same raw material, and different fractions were distinguished by high levels of specific phenolic compounds. Meanwhile, a similar pattern of phenolics was determined between fractions of lingonberry leaves and fruits obtained from the same fractionation steps, indicating different phenolics elution with solvents of a certain polarity.

### 3.4. Antioxidant Activities of Lingonberry Fractions 

Antioxidant activities of fractions of lingonberry leaves and fruits, including crude extracts, were variable (Table 3). The radical scavenging activity assessed by ABTS assay ranged between 1751.4 and 7832.7 μM TE/g DW, with the lowest and highest (*p* < 0.05) values determined in the crude extract of lingonberry fruits marked F1 and fraction of lingonberry leaves marked L4, respectively. A similar antiradical activity was observed between L2 and F2 fractions, and between L3, F3, and F4 fractions. In FRAP assay, L3 and F3 fractions surpassed all other fractions by the greatest (*p* < 0.05) reducing activity, followed by L4 and F4 fractions. Whereas the lowest (*p* < 0.05) reducing activity was found in the crude extract of lingonberry fruits marked F1, with almost eight and a half lower activity than average. FIC assay showed a similar trend to the results of ABTS and FRAP assays. The highest (*p* < 0.05) chelating activity was expressed in the fractions of L4 and F4, and the lowest (*p* < 0.05) one, approximately three times lower, in F1. There were no significant differences between the activity of L1, L2, and F3 fractions, while the activity of F2 was slightly higher (*p* < 0.05) than L2.

Antioxidant activity of crude extracts of lingonberry leaves and fruits or obtained fractions according to average values of all antioxidant assays were in the following ascending order: F1 < L2 ~ F2 < L1 < F3 ~ F4 < L3 ~ L4. All assays confirmed the lowest antioxidant activity of crude extract of lingonberry fruits, and the highest activity of fractions, enriched with catechins, flavonols, or proanthocyanidins, depending on the applied method and the predominant mechanism of action. It was found that the average antioxidant activity of fractions of lingonberry leaves, including crude extracts, was nearly one and a half times greater than the average antioxidant activity of fractions of lingonberry fruits.

### 3.5. Anticancer Activity of Lingonberry Fractions and Phenolic Compounds

**Figure 5:**
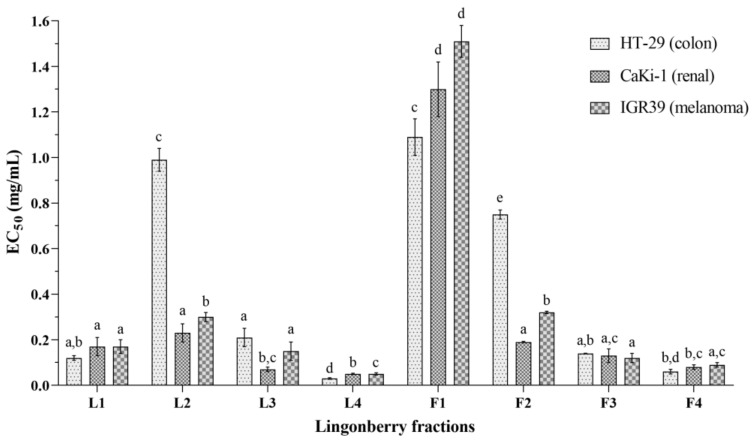
Anticancer activity of fractions of lingonberry leaves and fruits against different cancer cell lines. Bars marked with different letters indicate statistically significant differences (*p* < 0.05) within the same category.

Lingonberry fractions of leaves and fruits, including crude extracts, reduced the viability of malignant melanoma (IGR39), colon adenocarcinoma (HT-29), and clear cell renal cell carcinoma (CaKi-1) lines (Figure 5). In general, crude extract of lingonberry fruits marked F1 possessed the lowest (*p* < 0.05) anticancer activity against all three cell lines (EC_50_ varied from 1.1 to 1.5 mg/mL against different cell lines). Fractions L4 and V4, which were predominated with proanthocyanidins, were the most active ones (EC_50_ varied from 0.03 to 0.05 mg/mL and from 0.06 to 0.1 mg/mL, respectively, against different cell lines). A majority of tested lingonberry fractions were more active against renal cancer cells and showed lower activity against colon cancer cell line.

Fractions of lingonberry leaves were distinguished by about two times greater average values of anticancer activity compared to fractions of fruits. Anticancer activity of lingonberry fractions based on average values of anticancer activity against different cell lines was in the following anticancer activity ascending order: F1 < L2 ~ F2 < L1 ~ L3 ~ F3 < F4 ~ L4.

The results provided in Table 4 demonstrate anticancer activity of major phytochemical markers of different lingonberry fractions against malignant melanoma (IGR39), colon adenocarcinoma (HT-29), and clear cell renal cell carcinoma (CaKi-1) lines. Only two phenolic compounds (quercetin and procyanidin A2) reduced the viability of cancer cell lines by 50% at lower than 200.0 μM concentration. The highest anticancer activity—about two times higher compared to activity against colon cancer cell line—was found against renal cancer cell lines. Other phenolic compounds did not distinguish by high anticancer activity and did not reduce the viability of cancer cell lines by 50% at the highest tested concentrations in the range from 200.0 μM (procyanidin A1, C1) to over 1000.0 μM (arbutin).

### 3.6. Correlation Analysis

Since higher values of antioxidant activity (expressed as equivalents of standard antioxidants) indicate higher activity, and higher values of cell viability assay (expressed as EC_50_) indicate lower activity, strong negative correlations (*r* > −0.700, *p* < 0.05) between antioxidant and anticancer activities of lingonberry fractions were found. The strongest correlation (*r* = −0.955, *p* < 0.05) was found between FIC antioxidant activity and MTT assay against malignant melanoma cancer cell line.

Strong correlations between results of assays of antioxidant and anticancer activity and specific compounds in different fractions of lingonberries were also observed. Obtained antioxidant activities were strongly correlated with contents of flavonol aglycones in lingonberry fractions, with the strongest correlation between values of ABTS assay and quercetin contents in fractions of lingonberry leaves and fruits (*r* = 0.994 and *r* = 0.900, *p* < 0.05, respectively). Values of ferric reducing activity of different fractions were also well correlated (*r* > 0.700, *p* < 0.05) with contents of all flavonol glycosides, catechins, and procyanidins B1, B2, B3. On the other hand, greater ferrous chelating activity was related to higher contents of procyanidins C1, A1, A2 (*r* > 0.650, *p* < 0.05). A similar tendency of results was obtained when assessing correlations between values from MTT assays with phenolics in different fractions. It was found that higher content of quercetin in lingonberry fractions determined higher anticancer activity and lower EC_50_ values against different cancer cell lines. The strongest correlation (*r* = −0.974, *p* < 0.05) was found between values of MTT assay against clear cell renal cell carcinoma line and quercetin contents in fractions of lingonberry leaves, followed by the strong correlation (*r* = −0.939, *p* < 0.05) between values of MTT assay against colon adenocarcinoma cell line and quercetin contents in fractions of lingonberry fruits. Furthermore, strong correlations between values of MTT assay against all cancer cell lines and proanthocyanidins contents in fractions of lingonberry leaves and fruits were also observed, with the strongest correlation (*r* = −0.802, *p* < 0.05) between anticancer activity against melanoma cancer cell line and proanthocyanidins A1, A2, C1 contents in fractions of lingonberry leaves.

## 4. Discussion

Leaves and fruits of *V. vitis-idaea* are complex matrixes containing a body of phenolic compounds of various structural peculiarities. From the results, it is clear that extracts of lingonberry leaves can be characterized by high contents of arbutin, catechins, specific proanthocyanidins, and flavonols, whereas extracts of lingonberry fruits differ by the much lower content of arbutin, presence of anthocyanins, and greater diversity of phenolic acids. This is in accordance with the findings reported earlier [32,33]. Quantitative differences between crude extracts of lingonberry leaves and fruits—significantly higher levels of total identified phenolics in leaves, observed in the present material—can be explained as follows. Lingonberry fruits are highly loaded with sugars—glucose, fructose, and sucrose—resulting in a much lower contribution of other bioactive compounds, such as phenolics to the total mass of lingonberries [34].

Mentioned sugars were regarded to be removed from lingonberry fruits with the first water portion, because, after further elution with acidified 20% methanol, a significant increase in the content of phenolic compounds was observed. Major lingonberry anthocyanins—cyanidin-3-*O*-galactoside, cyanidin-*O*-arabinoside, and cyanidin-*O*-glucoside—which were identified in fraction F2, are responsible for the red color of lingonberry fruits and can be characterized by potential anti-ischemic, anti-inflammatory, and anti-hyperlipidemic effects [35,36]. The high content of benzoic acid, which was also observed in a 20% methanolic fraction, might contribute to the acidity of the lingonberries and be responsible for strong antimicrobial activity, as reported by previous researchers [37]. In line with the previous report, hydroxycinnamic acids can be regarded to be less abundant in lingonberry fruits, and only higher levels of chlorogenic, cryptochlorogenic, and *p*-coumaric acids were determined in the F2 fraction in the present work [12]. Since lingonberry leaves are not characterized by high amounts of sugars, the first step of fractionation of crude extract of leaves did not include an additional water-washing stage, which was applied in the case of crude extract of fruits, and led to a direct concentrating of arbutin as major phenolic of L2 fraction. A high level of arbutin in herbal raw materials is associated with urinary antiseptic and skin whitening properties, thus revealing the possible application of this fraction in cosmetics and pharmaceuticals [38].

In accordance with Amarowicz et al., 50% ethanol’s lesser polarity provided better separation of phenolics compared with methanol and resulted in fractions of lingonberry leaves and fruits (L3 and F3, respectively), which were partly free from major phenolic acids, anthocyanins, arbutin, and were distinguished by an abundance of catechins and various flavonols [39]. In comparison to L3, fraction F3 was characterized by the absence of nicotiflorin, 6”-*O*-acetylisoquercitrin, and lower or similar levels of all other flavonol glycosides, except by the content of quercitrin, which can be regarded as major flavonol of lingonberry fruits in line with results of Zheng and Wang [14]. Resveratrol, known as a strong antioxidant and potent anticancer agent, was determined at low levels in lingonberry fruits before, as well as in the present study in the fraction marked F3 [40,41].

One of the most relevant groups of compounds are proanthocyanidins. The abundance of them in herbal raw materials is highly associated with health-protecting properties [42]. The last fractionation step with the 70% acetone as the most effective purification solvent—strong hydrogen bond breaker according to Kennedy—yielded bounded fractions of lingonberry leaves and fruits (L4 and F4, respectively) enriched with proanthocyanidins [43]. Proanthocyanidins from lingonberry have been reported to have very high antioxidant or anticancer activity in several studies [15,44,45]. This statement ties well with our present findings because of the greatest free radical scavenging, ferric reducing, chelating activities, and anticancer effect determined in fractions F4 and L4. A number of authors investigated lingonberry proanthocyanidins profile and tentatively based on mass spectral data identified A-or B-type procyanidin dimers and oligomers [46,47,48]. However, the comprehensive analysis using standard substances of procyanidins B1, B2, B3, A1, A2, and C1 has never been reported in lingonberries so far. Fractions F4 and L4 were voided from the other compounds of phenolic origin (except quercetin) and was presumably constituted of oligomeric proanthocyanidins, which ensure strong antioxidant and anticancer activity. It can be assumed that there are much more unidentified polymeric proanthocyanidins in these fractions as lingonberry raw materials contain a complex of A-trimers and compounds with higher polymerization degrees [46].

Research on grape seed extract elucidated the numerous biological effects of proanthocyanidins and their potent antioxidant activity. Studies confirm their significance in cancer chemoprevention and inhibition [49]. Lingonberry raw materials as well contain proanthocyanidins, whose activities have not been comprehensively characterized yet. Uniformly standardized preparation is one of the challenges that result in controversial and hardly comparable biological effects. Grape seed extract consists mainly of B-type proanthocyanidins, whereas lingonberry raw materials contain both B and A-type linked proanthocyanidins with the unique patterns of A-type trimers. A-type proanthocyanidins with double chain linkage are specific to the *Vaccinium* genus and are expressed in greater amounts than single chain-linked B-type. Interlinkages of subunits increase the electron delocalization capacity of the phenyl radical and are important structural features for the activity [15,49]. Proanthocyanidins inhibit cell growth, disrupt cell cycle, and target abnormal cells to apoptotic death. Also, they are able to arrest the cell cycle, induce apoptosis in human colorectal cancer cells independently of their p53 status [49,50].

Our obtained fractions L4 and F4 were more active against HT-29 cell line compared to the grape seed extract, which is also rich in proanthocyanidins, as revealed by Kaur et al. Grape seed extract at 50 µg/mL concentration reduced the number of live HT-29 cells by only 20% after 48 h of incubation [51]. According to the present findings, the HT-29 cell line was the least susceptible to the anticancer effect of lingonberry fractions or standard substances. It can be explained by five times greater resistance to drug treatment of this cell line compared to other colon adenocarcinoma cell line—HCT116 [52]. Other researchers established that grape seed extract containing about 89% of proanthocyanins, was also more active in the HCT116 cell line (30 µg/mL of extract killed about 60% of cells already after 48 h) [53]. Interestingly, grape seed extract showed lower activity against melanoma cancer cell line A431 (cell viability was reduced by only ~20% at 50 and 100 µg/mL concentrations, while in our experiment L4 and F4 fractions reduced melanoma IGR-39 cell viability by 50% at 50–100 µg/mL concentrations after 72 h of incubation [54]. It could be explained by the proanthocyanidins specificity in different cell lines and time and dose-dependent effect.

Low anticancer activity of anthocyanins and the phenolic acids-rich fraction of lingonberry fruits (F2), especially against colon adenocarcinoma cell line, was consistent with McDougall et al., who noted that anthocyanins-rich fraction was considerably less effective, and anticancer activity of lingonberries was determined in the proanthocyanidins-rich fraction [55]. Angelova et al. found that non-anthocyanin fractions of Bulgarian lingonberry have well expressed inhibitory effect on survival of human cervical (HeLa) and breast (MCF-7) cancer cell lines, and suggested to us that future research should consider the potential anticancer activity of lingonberry against other non-studied cancer cell lines [44]. Our novel findings on low antioxidant activity of crude extract of lingonberry fruits, also of fractions, enriched with arbutin or phenolic acids, were in line with Bujor et al., who reported that the antioxidant activity of lingonberry fruits is significantly lower compared to stems or leaves, and with our previous study on antioxidant activity of standard substances of arbutin, ferulic, chlorogenic, and cryptochlorogenic acids [12,27]. This confirms the necessity of fractionation of lingonberry raw materials to enhance target phenolic levels and indicates that anticancer and antioxidant agents of lingonberries are retained mainly in the proanthocyanidins-bounded fractions.

Proanthocyanidins are not absorbed and they enter the large intestine where they start to be metabolized to simple phenolic compounds. Therefore, they can express their effects in an unaltered state [56]. Lingonberry anthocyanins-rich fraction can be metabolized to cyanidin glucuronides or parent compounds such as cyanidin-3-*O*-galactoside and cyanidin-3-*O*-glycosides and they can be detected in urine and serum [55,56,57]. Lehtonen et al. detected quercetin glycosides and glucuronides in urine and plasma after ingestion of lingonberry fruits [58]. Certain levels of proanthocyanidin dimers and trimers have been also detected in urine and plasma and may unchanged reach colonocytes, where they can modulate cellular processes. It can be presumed that phytochemical markers of lingonberry fractions can reach the target sites in renal and colon and express chemopreventive and chemotherapeutic effects [50].

Phenolic compounds can express antioxidant activity via multiple mechanisms such as direct scavenging of ROS, reducing the oxidative substances or stabilizing them, chelating metal ions, or modulation of endogenous antioxidant systems [4]. All these mechanisms can be interconnected with inflammatory processes and stages of carcinogenesis. Depending on the conditions, phenolic compounds can act as antioxidants or pro-oxidants, confirming their capabilities in redox modulation. Highly antioxidant capable phenolic compounds can disturb ROS-mediated carcinogenesis [5]. The greatest free radical scavenging, chelating, and different cancer cells viability reducing activities determined in the fractions (F4, L4), enriched with proanthocyanidins and slightly higher ferric reducing the antioxidant activity of fractions (F3, L3) with predominant phenolics groups of catechins and flavonols, indicates possible differences in the prevailing mechanism of action. This is in accordance with Csepregi et al., who pointed out that strong antioxidant activity, particularly determined by FRAP assay, is positively associated with the presence of catechol structure in ring-B and 3-hydroxyl group in ring-C of catechins and flavonols [59]. The strongest correlation between values of FIC assay and anticancer activity against malignant melanoma cancer cell line—also results of the previous study—indicates that chelating agents may play an important role in reducing the concentration of the catalyzing transition metals in ROS-mediated melanoma carcinogenesis [60]. The anticancer effect on lingonberry fractions was not related to the doubling time of cell lines. According to the data provided in a Swiss Bioinformatics Resource Portal Expasy, the HT-29 cell line has the lowest doubling time between tested ones, about 24 h, and the most sensitive cell line in our research was CaKi-1, which doubles in about 40 h. It could mean that the direct mechanism of action of substances found in lingonberry extracts and their fractions could not be or is not only related to the mitosis inhibition in cells [61].

Because of strong correlations between proanthocyanidins, quercetin levels, and antioxidant or anticancer activities in different fractions, these compounds can be considered as the major contributors to the biological activity of lingonberries. This was confirmed by our novel findings on cancer cell viability reducing activity of standard substances of major phenolics of lingonberries, which revealed the strongest anticancer activity of quercetin and procyanidin A2, as well as by our previous study, which showed the highest antioxidant activities of standard substances of procyanidins C1 and A2 [27]. It can be assumed that these particular compounds, or complex of dimeric-polymeric proanthocyanidins, are highly related to defense mechanisms against potentially harmful radical species and anticancer activity of lingonberries. Further and more detailed studies on phytochemical characterization and biological activity of different fractions, enriched with specific phenolics, are needed to examine the possible application of different lingonberry phenolics in the pharmaceutical industry.

## 5. Conclusions

The phytochemical analysis of crude extracts and phenolic fractions of lingonberries, by a validated analytical method with further evaluation of their free radical scavenging, reducing, and chelating activities, and anticancer activity against three cancer cell lines revealed large phytochemical diversity and highlighted predominant lingonberry phenolics. Out of them, quercetin and proanthocyanidins can be regarded as markers of antioxidant and anticancer activities of extracts of lingonberry leaves and fruits. These lingonberry compounds might be beneficial as promising chemo-active agents for alternative novel phytopreparations and nutraceutical products.

## Figures and Tables

**Table 1:** Content of phenolics (μg/g DW ± SD) in fractions of lingonberry leaves.

Peak No.	Identity	Fractions of Lingonberry Leaves			
		L1	L2	L3	L4
**1**	Arbutin	127,473.1 ± 988.3 ^a^	286,718.3 ± 2266.0 ^a^	ND	ND
**2**	Neochlorogenic acid	373.9 ± 8.4 ^b^	989.4 ± 5.7 ^b^	ND	ND
**4**	Procyanidin B1	1625.2 ± 20.9 ^c,d^	ND	8780.1 ± 415.2 ^a^	496.2 ± 27.9 ^a^
**5**	Procyanidin B3	9323.5 ± 222.4 ^e^	ND	56,120.3 ± 1881.8 ^b^	2709.4 ± 96.2 ^b^
**6**	Chlorogenic acid	2625.3 ± 123.5 ^f^	3384.8 ± 55.3 ^c^	ND	ND
**7**	(+)-Catechin	29,125.0 ± 227.6 ^g^	ND	170,419.4 ± 4222.2 ^c^	2615.8 ± 5.3 ^b^
**8**	Cryptochlorogenic acid	2861.8 ± 69.4 ^f^	2636.2 ± 24.9 ^c^	ND	ND
**9**	Procyanidin B2	4012.9 ± 120.5 ^h^	ND	23,370.0 ± 141.0 ^d^	6838.9 ± 69.8 ^c^
**12**	(−)-Epicatechin	5992.6 ± 47.0 ^i^	ND	32,353.2 ± 370.3 ^e^	5114.3 ± 89.3 ^d^
**15**	Procyanidin C1	3614.0 ± 143.1 ^h^	ND	ND	9840.1 ± 159.3 ^e^
**17**	2-*O*-Caffeoylarbutin	7724.5 ± 189.4 ^l^	ND	47,309.4 ± 1433.4 ^h^	ND
**18**	Procyanidin A1	12,969.4 ± 217.8 ^j^	ND	ND	50,580.9 ± 278.0 ^f^
**19**	Rutin	2362.3 ± 50.2 ^d,f^	ND	14,479.7 ± 44.8 ^f^	ND
**20**	*p*-Coumaric acid	706.5 ± 19.5 ^b,k^	475.5 ± 13.1 ^b^	236.5 ± 3.6 ^g^	ND
**21**	Hyperoside	5439.3 ± 120.5 ^i^	ND	33,594.0 ± 497.9 ^e^	ND
**22**	Isoquercitrin	1612.8 ± 71.8 ^c^	ND	10,263.1 ± 432.7 ^a^	ND
**23**	Procyanidin A2	2787.3 ± 77.6 ^f^	ND	ND	16,500.0 ± 467.2 ^g^
**26**	Nicotiflorin	289.3 ± 9.6 ^b^	ND	1596.6 ± 60.0 ^g^	ND
**27**	Reynoutrin	1329.1 ± 28.2 ^c,k^	ND	8421.4 ± 278.0 ^a^	ND
**28**	Guaiaverin	2353.7 ± 56.5 ^d,f^	ND	14,706.2 ± 404.2 ^f^	ND
**29**	Avicularin	5637.9 ± 42.3 ^i^	ND	34,465.1 ± 1142.6 ^e^	ND
**30**	Astragalin	230.4 ± 11.1 ^b^	ND	1302.4 ± 54.5 ^g^	ND
**31**	Quercitrin	3672.3 ± 134.0 ^h^	ND	23,107.4 ± 979.5 ^d^	ND
**32**	6”-*O*-Acetylisoquercitrin	97.0 ± 1.4 ^b^	ND	668.7 ± 24.5 ^g^	ND
**34**	Afzelin	108.6 ± 6.2 ^b^	ND	983.9 ± 24.2 ^g^	ND
**35**	Quercetin-HMG-rhamnoside	5355.0 ± 37.1 ^i^	ND	32,053.4 ± 812.7 ^e^	ND
**37**	Quercetin	157.0 ± 5.0 ^b^	ND	1031.1 ± 21.1 ^g^	1054.2 ± 3.1 ^a^
**39**	Kaempferol	NQ	ND	NQ	NQ
Total identified		239,859.8	294,204.2	515,261.7	95,749.8

Different letters represent statistically significant differences (*p* < 0.05) between phenolics within the same fraction; ND—not detected, NQ—not quantified (amount below LOQ).

**Table 2:** Content of phenolics (μg/g DW ± SD) in fractions of lingonberry fruits.

Peak No.	Identity	Fractions of Lingonberry Fruits			
		F1	F2	F3	F4
**1**	Arbutin	780.9 ± 12.4 ^a^	4698.8 ± 118.4 ^a^	ND	ND
**2**	Neochlorogenic acid	39.9 ± 0.8 ^b,c^	642.6 ± 21.6 ^b^	ND	ND
**3**	Protocatechuic acid	91.7 ± 5.3 ^c,d^	7023.4 ± 328.7 ^c^	ND	ND
**4**	Procyanidin B1	611.39 ± 18.5 ^e^	ND	19,014.8 ± 352.1 ^a^	1341.7 ± 24.8 ^a^
**5**	Procyanidin B3	424.8 ± 17.2 ^f^	ND	11,263.1 ± 82.7 ^b^	2941.9 ± 20.1 ^b,c^
**6**	Chlorogenic acid	222.4 ± 67.5 ^g^	17,761.3 ± 612.9 ^d^	ND	ND
**7**	(+)-Catechin	1243.8 ± 31.0 ^h^	ND	56,758.2 ± 1819.9 ^c^	2052.7 ± 71.0 ^d^
**8**	Cryptochlorogenic acid	144.1 ± 5.7 ^d^	8466.4 ± 171.1 ^e^	ND	ND
**9**	Procyanidin B2	222.8 ± 4.6 ^g^	ND	10,547.7 ± 226.5 ^b^	2665.6 ± 18.1 ^b^
**10**	Cyanidin-3-*O*-galactoside	319.0 ± 9.6 ^i^	51,065.6 ± 529.5 ^f^	517.36 ± 5.95 ^d,i^	NQ
**11**	Cyanidin-3-*O*-glucoside	49.1 ± 1.3 ^b,c^	7477.4 ± 338.6 ^c,e^	30.0 ± 0.8 ^i^	ND
**12**	(−)-Epicatechin	214.1 ± 28.3 ^g^	ND	10,853.8 ± 123.6 ^b^	626.7 ± 20.2 ^e^
**13**	Caffeic acid	15.3 ± 1.0 ^b^	284.3 ± 17.7 ^b^	ND	ND
**14**	Cyanidin-3-*O*-arabinoside	58.0 ± 7.7 ^b,c^	10,195.3 ± 493.4 ^g^	79.2 ± 0.3 ^i^	ND
**15**	Procyanidin C1	82.8 ± 2.0 ^c,d^	ND	ND	3230.7 ± 29.1 ^c,f^
**16**	Vanillic acid	16.5 ± 1.2 ^b^	693.6 ± 35.9 ^b^	ND	ND
**17**	2-*O*-Caffeoylarbutin	32.1 ± 0.8 ^b,c^	ND	1134.5 ± 49.7 ^d^	ND
**18**	Procyanidin A1	548.5 ± 16.8 ^e^	ND	ND	13,967.9 ± 334.7 ^g^
**19**	Rutin	121.7 ± 3.5 ^d^	ND	972.2 ± 42.1 ^d^	ND
**20**	*p*-Coumaric acid	81.9 ± 2.4 ^c,d^	4491.7 ± 157.7 ^a^	1177.9 ± 7.8 ^d^	ND
**21**	Hyperoside	228.3 ± 3.8 ^g^	ND	16,900.9 ± 388.3 ^e^	ND
**22**	Isoquercitrin	119.2 ± 4.6 ^d^	ND	6487.9 ± 184.8 ^f^	ND
**23**	Procyanidin A2	324.8 ± 10.4 ^i^	ND	ND	10,178.0 ± 31.1 ^h^
**24**	Sinapic acid	16.5 ± 0.1 ^b^	302.6 ± 2.3 ^b^	ND	ND
**25**	Ferulic acid	22.7 ± 1.8 ^b^	568.8± 5.4 ^b^	ND	ND
**27**	Reynoutrin	89.8 ± 4.7 ^c,d^	ND	4467.9 ± 69.0 ^g^	ND
**28**	Guaiaverin	41.6 ± 2.2 ^b,c^	ND	3221.4 ± 169.4 ^h^	ND
**29**	Avicularin	142.1 ± 4.4 ^d^	ND	11,499.6 ± 90.9 ^b^	ND
**30**	Astragalin	NQ	ND	311.8 ± 5.5 ^d,i^	ND
**31**	Quercitrin	584.1 ± 30.5 ^e^	ND	46,144.7 ± 535.8 ^j^	ND
**33**	Benzoic acid	1385.6 ± 57.0 ^j^	50,569.0 ± 350.6 ^f^	ND	ND
**34**	Afzelin	14.9 ± 1.0 ^b^	ND	1099.1 ± 31.8 ^d^	ND
**35**	Quercetin-HMG-rhamnoside	214.1 ± 13.9 ^g^	ND	13,859.8 ± 314.8 ^k^	ND
**36**	Resveratrol	NQ	ND	27.0 ± 0.6 ^i^	ND
**37**	Quercetin	298.5 ± 14.4 ^i^	ND	1229.6 ± 21.0 ^d^	3423.8 ± 42.4 ^f^
**38**	*trans*-Cinnamic acid	193.9 ± 1.3 ^g,d^	4376.0 ± 95.5 ^a^	ND	ND
**39**	Kaempferol	NQ	ND	87.0 ± 3.1 ^i^	93.2 ± 0.6 ^i^
Total identified		8996.5	168,616.6	217,685.3	40,522.3

Different letters represent statistically significant differences (*p* < 0.05) between phenolics within the same fraction; ND—not detected, NQ—not quantified (amount below LOQ).

**Table 3:** Antioxidant activities of different fractions of lingonberry leaves and fruits.

Fractions	μM TE/g DW (ABTS)	μM TE/g DW (FRAP)	µM EDTA/g DW (FIC)
L1	4977.6 ± 75.3 ^a^	2805.6 ± 14.4 ^a^	161.8 ± 3.4 ^a^
L2	4204.1 ± 186.6 ^b^	1807.2 ± 22.3 ^b^	126.4 ± 2.2 ^b^
L3	7338.4 ± 37.3 ^c^	5316.7 ± 169.6 ^c^	167.8 ± 4.3 ^a^
L4	7832.7 ± 68.4 ^d^	4750.3 ± 81.3 ^d^	184.6 ± 1.6 ^c^
F1	1751.4 ± 92.1 ^e^	404.9 ± 39.9 ^e^	57.6 ± 3.2 ^d^
F2	4116.2 ± 118.5 ^b^	2147.0 ± 50.1 ^f^	139.1 ± 2.8 ^e^
F3	6961.2 ± 124.2 ^c^	5404.8 ± 79.3 ^c^	165.8 ± 2.4 ^a^
F4	7152.7 ± 96.9 ^c^	4287.5 ± 106.2 ^g^	177.6 ± 3.3 ^c^

Values marked with different letters in the same column are significantly different (*p* < 0.05).

**Table 4:** Anticancer activity of phenolic compounds against different cancer cell lines.

Compound		EC_50_ (μM)	
	HT-29 (Colon)	CaKi-1 (Renal)	IGR39 (Melanoma)
Arbutin	>1000.0	>1000.0	>1000.0
Avicularin	>500.0	>500.0	>500.0
Quercitrin	>500.0	>500.0	>500.0
Quercetin	47.4 ± 2.0 *	40.8 ± 5.4 *	62.16 ± 9.4 *
(+)-Catechin	>500.0	>500.0	>500.0
Procyanidin A1	>200.0	>200.0	>200.0
Procyanidin A2	145.5 ± 9.6	63.0 ± 4.9 *	97.27 ± 4.4 *
Procyanidin C1	>200.0	>200.0	>200.0
Procyanidin B3	>500.0	>500.0	>500.0

Values marked with * indicate the highest (*p* < 0.05) anticancer activity within the same column.

## Supplementary Materials

The following are available online at https://www.mdpi.com/2076-3921/9/12/1261/s1, Table S1: HPLC-PDA method validation parameters of linearity, LOD, LOQ, and precision for phenolics, Table S2: HPLC-PDA method validation parameters of trueness for phenolics.
