# Supplementary material for: Phenolic Fractions from Vaccinium vitis-idaea L. and Their Antioxidant and Anticancer Activities Assessment
Source: Antioxidants (Basel). 2020 Dec 11;9(12):1261. doi: 10.3390/antiox9121261 (PMC7763140; doi:10.3390/antiox9121261)
Supplement: Supplementary file 1 [file antioxidants-09-01261-s001.pdf]

Table S1. HPLC-PDA method validation parameters of linearity, LOD, LOQ, and precision for phenolics.

Compound	Linearity range (μg/mL)	Calibration curve	Coefficient of determination (r^2)	LOD (μg/mL)	LOQ (μg/mL)	RSD, (%, n = 18)
Cryptochlorogenic acid	3.1–50.0	f(x) = 56700x - 91900	0.99993	0.11	0.33	0.5
Afzelin	1.6–50.0	f(x) = 36600x + 2650	0.99983	0.27	0.81	1.4
Arbutin	31.3–500.0	f(x) = 6430x + 31200	0.99996	0.56	1.70	0.1
Astragalin	1.6–50.0	f(x) = 42800x - 1820	0.99979	0.25	0.76	1.3
Avicularin	3.1–50.0	f(x) = 50100x + 6630	0.99972	0.24	0.73	0.5
Benzoic acid	3. 1-50.0	f(x) = 7700x + 723	0.99994	0.07	0.23	0.2
Caffeic acid	1.6-50.0	f(x) = 112000x - 24100	0.99974	0.37	1.25	1.5
(+)-Catechin	3.1–200.0	f(x) = 13000x - 3080	0.99999	0.31	1.02	0.2
Chlorogenic acid	3.1–50.0	f(x) = 48500x - 47200	0.99927	0.27	0.83	0.4
Cyanidin-3-*O*-arabinoside	1.6–100.0	f(x) = 47300x + 30900	0.99998	0.14	0.42	1.3
Cyanidin-3-*O*-galactoside	1.6–100.0	f(x) = 81200x + 54000	0.99999	0.06	0.18	0.2
Cyanidin-3-*O*-glucoside	1.6–100.0	f(x) = 41900x + 26700	0.99997	0.22	0.66	1.4
(–)-Epicatechin	3.1–50.0	f(x) = 13300x - 3010	0.99941	0.13	0.43	0.4
Ferulic acid	1.6–50.0	f(x) = 105000x - 7950	0.99994	0.47	1.42	1.0
Hyperoside	3.1-50.0	f(x) = 62900x - 1140	0.99973	0.19	0.58	0.3
Isoquercitrin	3.1-50.0	f(x) = 39100x + 2560	0.99995	0.13	0.39	0.6
Kaempferol	1.6–50.0	f(x) = 91700x - 5530	0.99996	0.05	0.15	1.7
Nicotiflorin	1.6–50.0	f(x) = 30600x + 7080	1.00000	0.05	0.14	1.2
Neochlorogenic acid	1.6–50.0	f(x) = 42500x - 15000	0.99970	0.32	0.97	1.3
p-Coumaric acid	1.6–50.0	f(x) = 179000x - 15000	0.99998	0.17	0.53	1.0
Procyanidin A1	3.1-50.0	f(x) = 14400x - 17000	0.99932	0.90	2.67	0.4
Procyanidin A2	3.1-50.0	f(x) = 17200x - 4410	0.99915	0.43	1.29	0.5
Procyanidin B1	3.1-50.0	f(x) = 8440x + 8740	0.99993	0.07	0.22	0.8
Procyanidin B2	3.1-50.0	f(x) = 7830x + 1430	0.99982	0.13	0.38	0.6
Procyanidin B3	3.1-50.0	f(x) = 8990x - 7070	0.99933	0.21	0.63	0.5
Procyanidin C1	3.1-50.0	f(x) = 7620x + 1850	0.99949	0.22	0.65	0.7
Protocatechuic acid	3.7–58.8	f(x) = 79400x + 5420	0.99998	0.04	0.14	1.1
Quercetin	1.6–50.0	f(x) = 74800x - 70600	0.99984	0.13	0.38	1.6
Guaiaverin	3.3–50.0	f(x) = 38600x + 2290	0.99996	0.12	0.36	1.2
6''-*O*-acetylisoquercitrin	1.6–50.0	f(x) = 34600x + 527	0.99999	0.05	0.15	1.5
Quercitrin	3.1–50.0	f(x) = 39500x + 3580	0.99961	0.23	0.71	0.5
Resveratrol	1.6–50.0	f(x) = 142000x + 7600	0.99999	0.16	0.47	1.7
Reynoutrin	1.6–25.0	f(x) = 34900x - 1790	0.99999	0.03	0.09	0.6
Rutin	3.1–50.0	f(x) = 30300x - 3050	0.99993	0.10	0.30	0.4
Sinapic acid	1.6–50.0	f(x) = 99000x + 22200	0.99988	0.08	0.25	1.1
trans-Cinnamic acid	3.1–50.0	f(x) = 137000x - 21400	0.99989	0.12	0.36	0.5
Vanillic acid	1.6–50.0	f(x) = 76400x + 19900	0.99999	0.02	0.08	1.7

Table S2. HPLC-PDA method validation parameters of trueness for phenolics.

Compound	Low concentration level		Medium concentration level		High concentration level	
	% Recovery	% RSD	% Recovery	% RSD	% Recovery	% RSD
Cryptochlorogenic acid	105.3	0.8	96.5	0.6	100.6	0.6
Afzelin	100.8	0.9	102.4	0.3	100.3	0.4
Arbutin	102.1	1.4	98.7	1.2	100.1	1.1
Astragalin	105.0	1.5	102.1	1.0	100.3	0.9
Avicularin	95.3	1.4	99.5	1.3	99.5	0.9
Benzoic acid	102.9	1.7	99.7	1.6	100.2	1.6
Caffeic acid	104.3	1.1	101.6	0.5	100.4	0.3
(+)-Catechin	107.0	1.0	99.1	1.4	100.0	1.2
Chlorogenic acid	103.1	1.1	98.5	0.4	99.1	0.2
Cyanidin-3-*O*-arabinoside	92.5	1.0	99.6	0.8	100.1	1.1
Cyanidin-3-*O*-galactoside	92.7	1.4	100.3	1.1	99.9	1.2
Cyanidin-3-*O*-glucoside	92.9	1.3	100.8	1.5	99.8	1.1
(–)-Epicatechin	95.4	1.2	95.9	1.6	97.8	1.3
Ferulic acid	107.5	1.1	98.1	0.9	100.1	0.3
Hyperoside	96.1	1.5	99.4	1.3	99.5	0.9
Isoquercitrin	95.1	1.4	101.0	1.2	99.8	0.7
Kaempferol	99.9	0.4	100.5	0.3	99.9	0.3
Nicotiflorin	98.1	1.3	99.9	1.1	100.0	0.5
Neochlorogenic acid	105.4	1.5	97.1	1.0	100.6	1.6
p-Coumaric acid	103.0	0.3	100.1	0.2	99.9	0.4
Procyanidin A1	107.1	1.6	90.1	1.7	90.3	1.2
Procyanidin A2	105.0	1.6	98.9	1.5	100.9	1.3
Procyanidin B1	91.8	1.6	99.9	1.4	99.8	1.4
Procyanidin B2	107.0	1.7	106.9	1.7	106.2	1.6
Procyanidin B3	105.9	1.6	100.6	1.7	100.8	1.0
Procyanidin C1	98.9	1.6	101.6	1.6	100.3	1.3
Protocatechuic acid	97.2	0.6	100.8	0.1	99.9	0.1
Quercetin	107.7	0.4	101.7	0.3	100.3	0.4
Guaiaverin	100.8	1.1	100.5	0.9	100.2	0.5
6''-*O*-acetylisoquercitrin	102.7	0.9	100.3	1.0	100.0	0.7
Quercitrin	101.6	0.8	101.7	0.6	100.5	0.6
Resveratrol	95.4	1.6	100.3	1.4	100.1	0.5
Reynoutrin	100.5	0.9	100.1	0.6	100.0	0.6
Rutin	100.9	1.4	100.3	1.0	100.2	1.3
Sinapic acid	92.2	0.6	101.0	0.7	99.7	0.4
trans-Cinnamic acid	103.1	0.9	98.2	0.8	100.3	0.6
Vanillic acid	98.2	0.2	99.9	0.2	99.9	0.1
